# Exploring the Impact of Augmented Reality in Children and Adolescents with Autism Spectrum Disorder: A Systematic Review

**DOI:** 10.3390/ijerph17176143

**Published:** 2020-08-24

**Authors:** Carmen Berenguer, Inmaculada Baixauli, Soledad Gómez, María de El Puig Andrés, Simona De Stasio

**Affiliations:** 1 Department of Developmental and Educational Psychology, University of Valencia, Avda. Blasco Ibáñez, 21, 46010 Valencia, Spain; carmen.berenguer@uv.es; 2 Catholic University of Valencia, Campus Capacitas, 46010 Valencia, Spain; inmaculada.baixauli@ucv.es (I.B.); mpuig.andres@ucv.es (M.d.E.P.A.); 3 Department of Human Studies, LUMSA University, 00193 Rome, Italy; s.destasio@lumsa.it

**Keywords:** autism, augmented reality, children, adolescents, outcomes, technology

## Abstract

Autistic Spectrum Disorder (ASD) is a neurodevelopmental condition characterized by persistent difficulties in communication and social interaction along with a restriction in interests and the presence of repetitive behaviors. The development and use of augmented reality technology for autism has increased in recent years. However, little is known about the impact of these virtual reality technologies on clinical health symptoms. The aim of this systematic review was to investigate the impact of augmented reality through social, cognitive, and behavioral domains in children and adolescents with autism. This study is the first contribution that has carried out an evidence-based systematic review including relevant science databases about the effectiveness of augmented reality-based intervention in ASD. The initial search identified a total of 387 records. After the exclusion of papers that are not research studies and are duplicated articles and after screening the abstract and full text, 20 articles were selected for analysis. The studies examined suggest promising findings about the effectiveness of augmented reality-based treatments for the promotion, support, and protection of health and wellbeing in children and adolescents with autism. Finally, possible directions for future work are discussed.

## 1. Introduction

Autistic Spectrum Disorder (ASD) is a neurodevelopmental condition characterized by persistent difficulties in communication and social interaction along with a restriction in interests and the presence of repetitive behaviors [1]. ASD has multiplied its prevalence by 4 in the last decade, with a rate of 1/68 in the USA [2]. The greatest increase has occurred in the subgroup of ASD without intellectual disability, that is, the high functioning autism or level 1 severity subgroup, with a prevalence of 62% [3]. Specifically, Blumberg et al. [4] indicates that children diagnosed after 2008 were more likely to have milder ASD. The steady growth of the prevalence of this disorder is partially due to a greater awareness among parents and professionals from health and educational services and to improvements in the diagnostic process, which has led to early detection and diagnosis [4].

This clinical condition has a significant impact on social life [5] and can have long-term negative consequences on different domains such as peer social interaction, cognitive abilities, daily life skills, academic achievement, and mental health [6,7]. Due to this diagnosis, children with this neurodevelopmental condition need effective treatments that improve not only the core symptoms of ASD but also the comorbid clinical presentations associated with the disorder.

Over the last decades, interventions based on the use of technology have risen exponentially as a real possibility of treatment to improve the health and quality of life of individuals with ASD and of their caregivers [8,9]. Augmented reality technology is a recent modality of virtual reality that expands reality images, combining virtual and real elements to create a mixed and interactive environment by adding virtual computer-generated information. In this way, artificial information about the environment and its objects can overlap in the real world [10].

Augmented reality (AR) technology has emerged as a means of effective treatment in different areas in the field of health: diagnosis, wellbeing promotion, and mental health treatment [11]. Regarding intervention programs, AR has been used to address the treatment of several neurodevelopmental disorders such as ASD. Specifically, in this area, AR has shown some advantages with respect to more traditional interventions, as it allows individuals with ASD to be treated in more ecological and realistic environments that may be manipulated and adapted to the specific and heterogeneous characteristics that children and adolescents with ASD exhibit [12]. Therefore, this kind of treatment would allow greater ecological validity in controlled environments and the skills learnt would be generalizable to other areas and daily life contexts [13,14].

Virtual reality (VR) provides sensory experiences in artificial environments through the computer, enabling virtual interactions. Augmented reality (AR), that constitutes a part of VR, allows an interaction in a physical world, which is not as artificial as in the case of VR [13]. Specifically, the interaction with VR generally requires the use of a specialized VR headset, which may be difficult to use for many children with ASD. AR technologies are simpler and more versatile because they use a wide range of devices, for example, tablets or Smartphones, better adapting the interaction to the real world. Research indicates that the applications based on AR allow children with ASD to engage in numerous multimodal interactions, facilitating the learning process of different abilities through the intervention programs, in comparison with VR [13,14].

Despite the promising results of the application of AR in interventions in children and adolescents with ASD, at present time, only two previous reviews [15,16] about ASD and AR have been carried out. A recent review focused on the use of AR for intervention in people with ASD [15]. Only 16 studies were identified, and many of them were presentations to congresses. Likewise, the review by Khowaja et al. [16] provided the state-of-the-art research regarding studies utilizing AR for children and adolescents with ASD to learn different skills. These recent reviews on the application of augmented reality (AR) technologies in ASD highlight the need for a better evidence base for the utility of AR in treatment and rehabilitation of individuals with special needs.

The objective of this systematic review is to investigate the impact of augmented reality on social, cognitive, and behavioral domains in children and adolescents with autism. The results of these interventions will be analyzed, trying to identify compliance with evidence-based quality criteria, according to the methodology proposed by Reichow [17]. Based on previous studies, we consider that AR applications can provide additional opportunities for implementing evidence-based treatments addressed to improve different skills that have been shown to be essential for individuals with ASD.

In addition, considering a recent meta-analysis, which includes only single-case studies examining how AR may help individuals with special needs, the current systematic review expands previous research by analyzing the potential effectiveness of AR technology in children and adolescents with ASD, covering both group and single-case studies.

To the best of our knowledge, this investigation is the first contribution that has carried out an evidence-based systematic review of studies that analyze the use of AR technologies in interventions promoting social, emotional, functional, and behavioral skills in children and adolescents with ASD.

## 2. Materials and Methods 

This systematic review was conducted in accordance with the Preferred Reporting Items for Systematic Reviews and Meta-Analyses (PRISMA) guidelines [18].

### 2.1. Search Strategy

Systematic searches were performed from March 2020 to April 2020 using the following electronic databases: PsycNET (PsycINFO), PubMed, Education Resources Information Centre (ERIC), Scopus, Web of Science Core Collection, and Psychology and Behavioral Sciences Collection (EBSCO). These databases were selected since they each cover a large part of the relevant literature on these topics. Searches were carried out using Boolean operators AND/OR by the following combination of keyword descriptors “autism”, “ASD”, “Asperger” “augmented reality”, “AR”, “mixed reality” “treatment”, “intervention”, “training”, “rehabilitation”, “education”, or “remediation.”

The title and abstract of the studies were screened following inclusion/exclusion criteria. Furthermore, a hand-search of the reference sections of relevant previous reviews along with reference lists of studies meeting the inclusion criteria was also conducted. Contributions of the following types were not included: reviews, abstracts, notes, protocols, letters, and editorials. The searches were limited to peer-reviewed studies where the authors carried out evaluation of the impact of augmented reality-based treatment in subjects with autism spectrum disorder. Since the advancement in AR technology has been increasing in the last decade, recent articles (from 1 January 2010 to 28 April 2020) were considered in the systematic review. Necessary data that were not included in the study were requested from the authors. Only studies in English were reviewed during the search.

### 2.2. Exclusion and Inclusion Criteria

The identified studies were included in accordance with the PICO model [19] in order to select the relevant research question in the selection criteria: P—population (children and adolescents (4–18 years old) had to have a diagnosis of autism spectrum disorder (ASD) or Asperger, I—intervention (the study was required to report an AR-based treatment or intervention), C—control group (versus non-AR-based treatment, children’s condition before AR-based treatment, and without treatment), and O—outcome (the main outcomes obtained). 

Additional criteria specified by the authors were the following: the Autism diagnosis should be established according to International Classification of Diseases (ICD) [20]; the Diagnostic and Statistical Manual of Mental Disorders (DSM) [1]; or the Autism Diagnostic Observation Schedule (ADOS) [21], the Revised Autism Diagnostic Interview (ADI-R) [22], or other validated caregiver questionnaires of ASD symptoms (e.g., Childhood Autism Rating Scale (CARS)—Schopler et al. [23]); the intervention design considered single-case studies or group research designs; and the studies have been published ideally in peer-reviewed journals or accepted for publication and were published in English.

Therefore, articles were excluded if (1) studies did not include children or adolescents with Autism Spectrum Disorder (ASD), (2) studies did not use AR technology-based therapies as interventions, (3) studies were not a primary research report (e.g., reviews, abstracts, notes, protocols, letters, and editorials), or (4) outcomes on patients were not reported.

### 2.3. Screening Process

The first two authors independently searched the literature and reviewed all the studies. Data were verified for accuracy and completeness by the rest of the authors. The screening process consisted of a title and abstract screening as well as a full-text screening based on the PICO criteria. If a study met all predefined eligibility criteria, it was included in the review. A total of 90% initial agreement was achieved on the database search. Any division of opinions was resolved by consensus among the authors of this work. This process was repeated until 100% agreement on the inputted data was reached. 

### 2.4. Methodological Quality Evaluation

Quality evaluation of the studies included in this systematic review was assessed using the evaluative method provided by Reichow [17]. This method is best suited to evaluating empirical research on specific interventions for individuals with autism spectrum disorders. Additionally, this method is equally appropriate in the evaluation of studies that used either single-subject or group comparison designs. According to Reichow’ [17] framework, the evaluative method involved a comprehensive protocol implemented across three stages. The first stage consists of evaluating the quality of each study using primary quality indicators: (1) information on participant characteristics, (2) definition of independent variables and dependent variables, (3) baseline conditions, and (4) visual analyses of the data. Secondary quality indicators include the use of interobserver agreement, blind raters, the calculation of the Kappa statistic, fidelity, generalization or maintenance, and social validity. Although secondary indicators are important, they are not considered necessary for determining the validity of a study. Each indicator was rated as either ‘‘high quality’’ (H), ‘‘acceptable quality’’ (A), or ‘‘unacceptable quality’’ (U). Lastly, studies were synthesized using a scoring criterion with studies receiving a categorization of ‘‘strong’’, ‘‘adequate’’, or ‘‘weak. Finally, studies were aggregated based on the number of participants in studies that used a single-subject research design and on the number of studies in group comparison designs who received effective treatment in studies categorized as ‘‘strong’’ or ‘‘adequate’’.

The above evidence-based practice (EBP) formula was applied per intervention to determine all possible combinations of evidence:

(GroupS*30) + (GroupA*15) + (SSRDs*4) + (SSRD_A_*2) = Z (1)

where GroupS is the number of studies conducted using group research designs earning a strong rating, GroupA is the number of studies conducted using group research designs earning an adequate rating, single subject research designs (SSRDs) is the number of participants for whom the intervention was successful from SSRD studies earning a strong rating, SSRD_A_ is the number of participants for whom the intervention was successful from SSRD studies earning an adequate rating, and Z is the total number of points for each study with 60 points indicating ‘‘established (EBP)’’ and with >30 points indicating ‘‘probable EBP’’ [17]. 

## 3. Results

### 3.1. Study Selection

**Figure 1:**
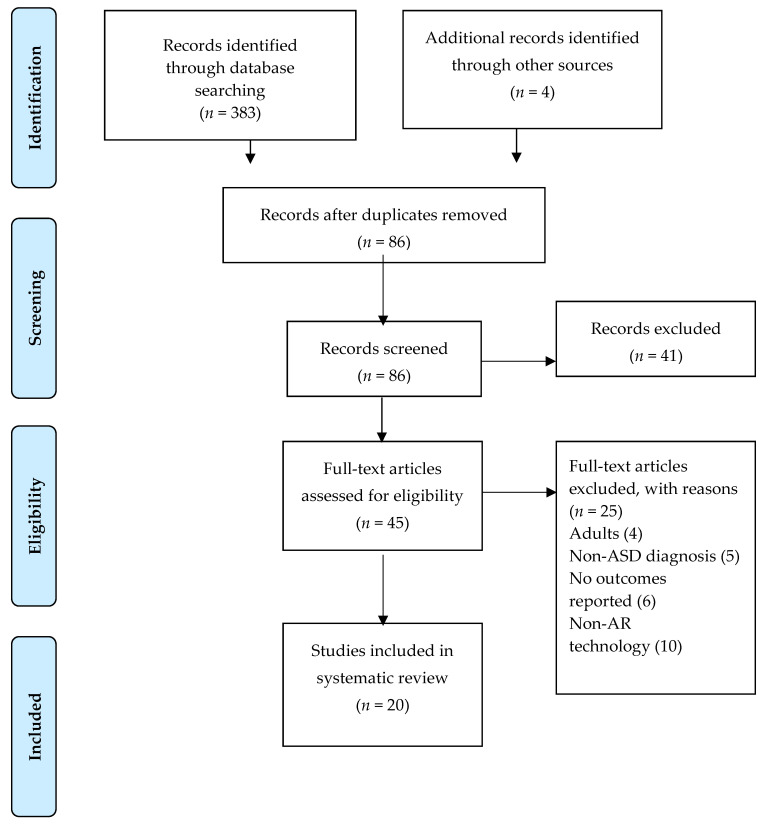
Preferred Reporting Items for Systematic Reviews and Meta-Analyses (PRISMA) flowchart of the study selection process for this systematic review.

The search yielded 387 articles distributed over time, as shown in Figure 1. After the removal of duplicates, 86 titles remained. Of these, 41 studies were excluded after title and abstract screening, as they did not fulfill the eligibility criteria. Of the remaining 45 studies that were subjected to full-text screening, 25 did not meet the inclusion/exclusion criteria. Specifically, four studies were excluded afterwards as they included adults (>18) in the sample of participants, five studies were excluded as they did not report an ASD diagnostic evaluation with a validated measure, six studies were excluded as they did not report clear outcomes, and finally, ten studies were excluded as they included virtual technology that were not AR technology. 

### 3.2. Study Characteristics

All included studies were scientific studies analyzing the effect of augmented reality-based interventions to improve different outcomes in children and adolescents with ASD. They were published in English between 2010 and 2020 (Table 1). 

This review analyzed and selected twenty studies [24,25,26,27,28,29,30,31,32,33,34,35,36,37,38,39,40,41,42,43]. Thirteen were single subject designs [24,26,27,28,30,31,32,36,37,38,39,40,41]. Five of them were multiple baseline design [24,26,27,31,32], and seven were group design studies [25,29,33,34,35,42,43]. The total number of participants among all the analyzed studies was 247, and 168 were children and adolescents with ASD. Their ages ranged from four to 18 years, with an average of 9.7 years. Regarding the gender of the sample, the total percentage of males was 65.8%. Furthermore, fifteen studies reported a clinical sample with an IQ > 70 [24,25,26,27,29,30,31,32,36,37,38,39,40,42,43], three studies reported a clinical sample with a low IQ (<70) [28,33,34], and two studies did not specify the IQ of the ASD participants [35,41]. All studies analyzed included augmented reality in their intervention process. 

Regarding the technology used in the different interventions, three studies were carried out with a smartphone [28,34,42], six studies used smartglasses [30,36,38,39,41,43], and eleven studies used multiple devices (augmented reality device with screens and helmet, computer games, or computer tablets). In the study by Cihak et al. [24], augmented reality application was downloaded onto an iPod and it allowed users to create an augmented reality experience by matching a user-created visual marker to trigger user-created digital content. The study by Bai et al. [25] used the metaphor of a mirrored view of reality enriched with AR augmentations. Therefore, it allows users to interact with the system without wearing or holding the display equipment and to manipulate physical toys with both hands. In the studies by Chen et al. [26] and Chen et al. [27], an AR system was designed using an augmented mirror through which users could see themselves with virtual 3-D facial expressions. In the study by Antao et al. [29], the intervention consisted of using a computer game (“MoviLetrando”) that uses the concept of projection-based AR with a webcam and created mirror images so that participants could see themselves on the screen. The studies by Lee et al. [31] and Lee et al. [32] used two different computer simulation programs: Concept mapping (CM) training system and Kinect Skeletal Tracking (KST) system, respectively. CM was design with AR interactive technology to teach children with ASD how to better comprehend social relations and to learn appropriate greeting responses. The augmented reality concept mapping (ARCM) training system was used as an instructional scaffold. Similarly, the KST system was combined with AR technology, and it was used for role-playing by trainers and children with ASD. The children with ASD saw 3-D virtual characters (classmates, teachers, neighbors, and salesclerks) animated by the trainers in 3-D contextual backgrounds on a screen. 

Magrini et al. [33] created an interactive augmented multisensory system (SIDOREMI), where the ASD child with fine motor difficulties moved in front of a screen driven by their parent and was stimulated to make a series of moves (repeat the movements, guess the movements, or connect the dots) thanks to several interactive modes. Farr et al. [35] used an AR environment, the Augmented Knight’s Castle (AKC) playmobil set, consisting of three base units that are wirelessly connected to a system server. Finally, Nazaruddin et al. [37] used books with a pop-up augmented reality format and Chung et al. [40] used AR videogames that most directly represented players in the digital game environment: ‘‘Fruit Ninja Kinect”, in which players use their silhouettes to cut fruit thrown into the gaming field, and ‘‘Kinect Party’’, in which real-life images are altered for entertainment purposes. 

**Table 1:** Main characteristics of the selected studies (*N* = 20).

Reference	Participants: *N*, Mean Age (MA) or Range, % Males, and IQ	Study Design	Technology/ Evaluation	Dependent Variables	Main Results
Cihak et al. [24]	ASD (3), 6–7 years, 100% males, IQ (70–75)	Single subject multiple baseline design	AR picture prompt to trigger a video model clip	Functional life skills (number of steps completed independently for brushing teeth)	AR was an effective tool for teaching chain tasks
Bai et al. [25]	ASD (12), MA (6.8 SD 5.5), % males (83%), IQ > 70	Within-subject experiment with two conditions: AR and non-AR	Set of AR props, video analysis, and parent and participants questionnaire	Elicit pretend play engagement	Significantly higher frequency and duration of pretend play in the AR condition and participants are more engaged
Chen et al. [26]	ASD (6), MA (11.5), males (83%), IQ 103.6 (9.3)	Single subject multiple baseline design	AR video modelling and storybook	Understand facial emotions and social expressions	Significant improvement in social/emotional awareness
Chen et al. [27]	ASD (3), MA (12.2), males (100%), IQ 101 (9.2)	Single subject multiple baseline design	AR-based self-facial learning system	6 basic facial expressions	Significant improvement in emotion recognition
Escobedo et al. [28]	ASD (12), MA (5.1 SD 0.9), males n.r., IQ low	Single subject design	AR smartphone	Selective and sustained attention	Improvement of both attention skills
Antão et al. [29]	ASD (48), MA (11, SD 5), males (89%), IQ > 70 TD (48), MA (11.8, SD 5.2) males (68%)	Group design	AR computer game “MoviLetrando”	Reaction time (RT)	ASD: significant improvement in RT after AR task
Liu et al. [30]	ASD (2), 8–9 age, males (100%), IQ > 70	Single subject design	AR smartglasses and parent questionnaires	Social interaction	Improvement in nonverbal communication, eye contact, and social engagement
Lee et al. [31]	ASD (3), MA (8.8) 67% males, IQ (93.3, SD 4.6)	Single subject multiple baseline design	AR CM training system and social story test	Social interaction	AR CM training system improved social relationships
Lee et al. [32]	ASD (3), MA (8.1) 67% males, IQ (102.3, SD 0.6)	Single subject multiple baseline design	KST System with AR technology and social story test	Social interaction	AR-KST System intervention improved significantly autism social interaction
Magrini et al. [33]	ASD (10), 5–7 years, males (100%), IQ low	Within-subject experiment with two conditions: AR (5 subjects) and non-AR (5 subjects)	AR system with multisensory experience	Fine Motor skills	Significant motor improvement in AR condition
Lorenzo et al. [34]	ASD (11), MA (4, SD 1.2), males (91%), IQ low	Within-subject experiment with two conditions: AR and non-AR	AR smartphone and questionnaires	Social interaction	Nonsignificant improvement between groups
Farr et al. [35]	ASD (12), MA (11.2), males (% 42%), IQ n.r.	Within-subject experiment with two conditions: AR and non-AR	AR Knight’s Castle (AKC) play	Social interaction	AR condition showed more social behavior interaction than non-AR
Sahin et al. [36]	ASD (8), MA (11.7 SD 3.3), males (88%), IQ > 70	Single subject design	AR smartglasses and questionnaires	Social interaction	Positive social experience after AR
Nazaruddin et al. [37]	ASD (4), 6–7 years, 50% males, IQ > 70	Single subject design	Augmented reality book and teacher questionnaire	Attention skills	AR book was able to increase focus and recognition of objects
Keshav et al. [38]	ASD (1), 13 years old, 100% males, IQ > 70	Single subject design	Empowered BrainAR Smartglasses and Social Responsiveness Scale-2 (SRS-2)	Social interaction and social communication skills	Improvement in SRS-2 social communication, motivation, and restricted and repetitive behavior subscales; improvements in verbal and nonverbal skills
Vahabzadeh et al. [39]	ASD (4), MA (7.5), male (100%), IQ > 70	Single subject design	Empowered brain, AR-computerized smartglasses, and Aberrant Behavioral Checklist (ABC)	Socioemotional and behavioral effects	Improvement in irritability, hyperactivity, and social withdrawal in a sample of students with ASD
Chung et al. [40]	ASD (3), 6–12 years old, males (100%), IQ > 70 non-ASD siblings (3), 6–12 years old, males (100%), IQ > 70	Single subject design	AR video games sessions	Social communication and joint positive affect	AR condition showed more joint positive affect and increased reciprocal communication
Soares et al. [41]	ASD (4), 8–12 years old, males (75%), IQ n.r. (low)	Single subject design	AR-based cardboard head-mounted display	Social interaction and facial processing	Better facial processing after use of AR cardboard display and EF implications
Escobedo et al. [42]	ASD (3), MA (10.1, SD 0.9), IQ > 70, non-ASD (11), 8–11 years old, IQ > 70	Group design	Mobile augmented reality application and selfreports	Social interaction	AR mobile application increased the number social interactions in ASD group
Nag et al. [43]	ASD (16), MA (12.1, SD 3.3), IQ (102.7, SD 19.5) non-ASD (17) MA (11.5, SD 2.4), IQ (108.9, SD 9.5), males n.r.	Group design	AR smartglasses task	Emotion recognition	AR task contributed to differentiating and classifying gaze and emotion recognition patterns between ASD and non-ASD groups

ASD: Autism Spectrum Disorder; AR: augmented reality, n.r.: not reported, SD: standard deviation, EF: executive functions, N: number, KST: Kinect Skeletal Tracking, CM: concept map, IQ: *intelligence quotient.*

### 3.3. Main Outcomes

Regarding to the effectiveness of the studies, 19 of the 20 studies stated that application of the AR-based treatment resulted in improvement of at least one of the aims addressed. However, one study specified that the results obtained did not show significant differences in the application of methods with or without augmented reality, although it seemed that AR improved the focus of attention of the children as well as their motivation. The authors suggested that changes in the design as well as in the measures could offer evidence based on results [34].

The main focus of most of the studies was on social interaction abilities (*n* = 11, 55%), including social communication, social interaction tasks, motivation, or social engagement, with promising outcomes [30,31,32,34,35,36,38,39,40,41,42]. A total of three studies (15%) aimed to improve attention skills (selective and sustained attention, and reaction time) [28,29,37]. Four studies (20%) provided an augmented visual indicator which improved emotion recognition and correct identification of facial expressions in children and adolescents with autism [26,27,41,43]. One study aimed to promote open-ended pretend play for young children with ASD [25]. Another study was oriented to teaching functional life skills through structured learning related to teeth cleaning [24]. Lastly, only one study was focused on improving gross and fine motor skills and on the enhancement of imitative aspects to foster social interaction and personal autonomy in children with autism by means of an AR prototype [33]. Table 2 shows the classification of studies by each domain or dependent variable. 

### 3.4. Methodological Quality Evaluation

The research strength of all included studies was calculated in accordance with Reichow’s [17] criteria. Table 3 provides a summary of the strength ratings for each study included in this systematic review. Thirteen studies (65%) received an adequate rating, seven studies (35%) received a weak rating, and none of the included studies that used an AR intervention were rated as strong. The evidence base for augmented reality interventions was calculated. This yielded a Z score of 53, indicating that augmented reality interventions could be categorized as probable in evidence-based practice [17].

## 4. Discussion

The aim of this review was to carry out an evaluation of the effectiveness of AR technologies on different domains as a result of the intervention process in children and adolescents with ASD. Likewise, this review aimed to identify compliance of the studies analyzed with evidence-based quality criteria according to the methodology proposed by Reichow [17]. Furthermore, this review focused on studies published over the last ten years, as AR is a relatively recent technology and, therefore, has great growing potential. With this purpose, 20 studies were selected after applying the determined eligibility criteria. 

Concerning quality of the studies, 13 of them [24,25,26,27,29,30,31,32,33,34,35,39,43] met the EBP criteria and seven did not reach these standards [28,36,37,38,40,41,42]. Eleven investigations focused on improving social information processing through activities addressed to evaluate social interactions, social motivation, or social communication, particularly related to participants’ pragmatic abilities. Six of these studies (54.5%) [30,31,32,34,35,39] met the EBP criteria, and 5 (45.4%) [36,38,40,41,42] did not meet the EBP criteria. In terms of results, only one of the studies did not reach conclusive and significant findings [34]. It is important to highlight that 72.7% of participants of the studies that analyzed social interactions were children and adolescents with high-functioning autism. 

In addition, 4 of the works explored how to increase facial and emotion recognition processing abilities through different tasks using varied applications of AR technologies. With respect to the 3 works that aimed to increase participants’ sustained or selective attention, only one met the EBP criteria [29]. Finally, other studies that addressed various domains such as emotion recognition, pretend play, functional skills, or motor skills also met quality standards [24,25,26,27,33,43]. For example, the study by Magrini et al. [33] aimed to improve fine-motor abilities in a sample of participants with low-functioning autism and dysgraphia. In comparison with the control group, children that participated in the experiment showed improvements in balance and hand movements. Moreover, a high level of acceptance of the VR platform was reported by the children and their parents. Another study [24] managed to incorporate a functional task like brushing teeth, with the aim of improving daily living skills in a heterogeneous sample of 12 children 4 to 7 years old. Similarly, in the study by Bai et al. [25], the AR experiment results confirmed that the AR system could help participants carry out pretend play more frequently, maintain longer pretend play duration, and keep their play ideas more consistent with suggested themes. 

In general, the majority of the analyzed studies demonstrated the beneficial effects of applying AR technology to improve diverse cognitive and emotional processes, social communication, theory of mind abilities like facial emotion recognition, attention, as well as functional and motor abilities. In effect, persistent difficulties in social communication and social interaction across multiple contexts as manifested by restrictions in social-emotional reciprocity, in developing and understanding relationships, or in nonverbal communicative behaviors used for social interactions [1] represent core characteristics of ASD. This symptomatology along with the problems associated with such a heterogeneous and complex disorder like ASD drives the need for specialized, individualized, and evidence-based interventions [44]. Thus, in the last decade, technological interventions addressed at children and adolescents with ASD have increased as a complement to cognitive-behavioral treatments based on observational learning proposed by Bandura [45].

From a qualitative perspective, these findings support the claim that the use of AR can provide a meaningful and enjoyable experience. In fact, many of the studies included in this review [24,25,26,27,33] report that AR applications not only promote social skills and new ways of learning among individuals with ASD but also offer them an engaging and cognitively demanding experience. The use of AR makes them feel more motivated and helps them understand information. Specifically, the majority of participants of the studies included in this review faced the experimentation with enthusiasm and, during the sessions, showed improvements in attention and response time [28,29,37]. Additionally, parents reported a high level of satisfaction with the different AR applications [40]. In general, the literature evidences that caregivers and teachers reported that children with ASD improved social interactions through advancements in nonverbal communication, social engagement, and eye contact while using AR technology [11,13].

Findings indicate that AR technology is an effective instructional strategy for teaching a multitude of behaviors in real-world settings for children and adolescents with autism [14]. AR applications can contribute to the way individuals with ASD learn daily life skills as well as can facilitate individuals’ understanding of social communicative behavior, enhancing attentional capacities and contributing to the recognition of facial emotions, among other advantages. The higher degree of realism provided by AR plays a key role in promoting a wide range of abilities that facilitate the autonomy and quality of life of children with ASD, allowing further approximation to the interactions with the real world. 

Studies have identified the advantages provided by computerized learning of different functional and social skills [11,14]. Specifically, among the strengths of the use of technology in interventions addressed at children with ASD, the possibility to clearly define tasks, to keep the focus of attention, to minimize distractions, and to facilitate personal skills and strategies with a tool that can be used for many applications is noteworthy. Another important advantage of the interventions based on AR technology is the reduction of social demands, sometimes unpredictable, that may be problematic for individuals with ASD. Likewise, the multimodal component provides an extensive multi-sensory experience (giving opportunities for users to use touch screens, sensors, cameras, and visual and audio cues), which is recommended in interventions directed at the population with ASD. However, caution must be taken with the use of programs based on technology like AR because there is a risk of more social isolation of children/adolescents with ASD. Another possible disadvantage is the lack of generalization of the benefits obtained after treatment.

AR is a kind of virtual reality technology that provides the individual with a mixed interactive experience, in other words, real and virtual, in an environment where it is possible to learn new behaviors and their generalization. Empirical studies that have analyzed at the moment the strengths of this technique with individuals with autism show promising results. Despite there being still scarce empirical investigations that the EBP criteria, the findings of this review suggest that AR techniques may be an effective complement in the field of cognitive-behavioral interventions in children and adolescents with ASD.

To our knowledge, this is the first review dealing with the possible effectiveness of AR techniques in children and adolescents with autism, based on empirical studies, both single-based and group studies. 

### Limitations

The main limitations of this study relate basically to the reduced number of high-quality designs carried out so far. Currently, as AR is a relatively recent technique, there are a lot of studies presented as communications and/or posters at international congresses and much less empirical studies of high-quality methodology. Other shortcoming of this review relate to the sample characteristics, as the majority of the included works that met the inclusion criteria had samples of children and adolescents with high-functioning autism. Despite the increasing rate of this subgroup of children with ASD [2], it would be necessary to analyze more studies that take into account a greater heterogeneity of the disorder in order to generalize the results. In the same vein, the reduced samples of many of the studies analyzed compromise the production of relevant results. Likewise, AR is a wide concept that employs diverse and multiple devices applied in intervention programs. In this review, only limited applications of AR are considered. Consequently, future research will have to consider application of more devices and applications (APPs) of AR related to the treatment of individuals with ASD in order to be able to determine their extent and effectiveness. 

Lastly, the lack of longitudinal investigations prevents us from knowing if these technologies may really help children with ASD improve social interactions or emotion recognition over time and in different developmental contexts. 

## 5. Conclusions

Based on the results obtained, AR technologies seem to have a positive effect on improving different domains such as social interaction, social communication skills, verbal and nonverbal communication, facial emotion recognition procedures, attention skills, or functional life in children and adolescents with autism.

The development of AR applications, computer games, tablet games, video games, or AR interactive books can be beneficial in the treatment process of this developmental disorder. In the studies reviewed, beneficial results were obtained in social interaction, emotion recognition, attention skills, and functional learning with AR technology. This approach may be especially suitable for the population with ASD, considering several factors. First, AR is based on strengths usually reported in ASD, like a visual learning style or a preference for visually displayed information [46]. Furthermore, some of the individuals with ASD may show a natural propensity to utilize digital tools, show a fondness for electronic media, and enjoy game-like elements, and this type of technology can help to reduce the stress that real-world social situations often produce on individuals with autism [47]. Therefore, AR allows the design of attractive learning tools which help children with ASD stay focused, promoting engagement with the activities, on-task behavior, motivation, and positive emotions that enhance the learning process [28,48]. Likewise, caregivers’ perceptions on AR reveal its high tolerability, usability, and engagement [30].

While it is true that more studies are required with better designs that have higher methodological quality and more significant results, we can state that this is an expanding field of research, where most of the publications are pilot studies or protocols of study and are exhibitions of technological development processes. Nonetheless, the studies suggest that it is necessary to expand the research and to carry out more elaborate scientific designs with representative samples that test AR applications under controlled conditions.

The increasing population of children with ASD requires interventions addressed to deal with their varied needs while maximizing their potential. Professionals must make informed decisions about which interventions are evidence-based and, then, to adjust them to the particular profile of each individual child. AR technologies seem to fit with the learning style of children with ASD and their interest in visual stimuli. This review contributes to the knowledge of the effectiveness of incorporating AR elements in interventions aimed at improving different areas of functioning in children and adolescents with ASD. The results are promising but more high-quality research based on rigorous methodologies is needed.

## Figures and Tables

**Table 2:** Dependent variables for included studies.

Reference	Elicit Pretended Play	Emotion Recognition	Functional Life Skills	Attention Skills	Social Interaction	Motor Skills
Cihak et al. [24]			X			
Bai et al. [25]	X					
Chen et al. [26]		X				
Chen et al. [27]		X				
Escobedo et al. [28]				X		
Antão et al. [29]				X		
Liu et al. [30]					X	
Lee et al. [31]					X	
Lee et al. [32]					X	
Magrini et al. [33]						X
Lorenzo et al. [34]					X	
Farr et al. [35]					X	
Sahin et al. [36]					X	
Nazaruddin et al. [37]				X		
Keshav et al. [38]					X	
Vahabzadeh et al. [39]					X	
Chung et al. [40]					X	
Soares et al. [41]		X			X	
Escobedo et al. [42]					X	
Nag et al. [43]		X				

**Table 3:** Summary of the strengths of each study.

Reference	Strength Rating (Reichow [17])
Cihak et al. [24]	Adequate
Bai et al. [25]	Adequate
Chen et al. [26]	Adequate
Chen et al. [27]	Adequate
Escobedo et al. [28]	Weak
Antão et al. [29]	Adequate
Liu et al. [30]	Adequate
Lee et al. [31]	Adequate
Lee et al. [32]	Adequate
Magrini et al. [33]	Adequate
Lorenzo et al. [34]	Adequate
Farr et al. [35]	Adequate
Sahin et al. [36]	Weak
Nazaruddin et al. [37]	Weak
Keshav et al. [38]	Weak
Vahabzadeh et al. [39]	Adequate
Chung et al. [40]	Weak
Soares et al. [41]	Weak
Escobedo et al. [42]	Weak
Nag et al. [43]	Adequate
